# Micronutrients and Markers of Oxidative Stress and Inflammation Related to Cardiometabolic Health: Results from the EHES-LUX Study

**DOI:** 10.3390/nu13010005

**Published:** 2020-12-22

**Authors:** Maria Ruiz-Castell, Gwenaëlle Le Coroller, Jean-Francois Landrier, Djedgiga Kerkour, Bernard Weber, Guy Fagherazzi, Brice M. R. Appenzeller, Michel Vaillant, Torsten Bohn

**Affiliations:** 1 Department of Population Health, Luxembourg Institute of Health, 1A-B, rue Thomas Edison, L-1445 Strassen, Luxembourg; djedjiga.kerkour@outlook.com; 2 Competence Centre for Methodology and Statistics, Department of Population Health, Luxembourg Institute of Health, 1A-B, rue Thomas Edison, L-1445 Strassen, Luxembourg; Gwenaelle.LeCoroller@lih.lu (G.L.C.); Michel.Vaillant@lih.lu (M.V.); 3 Aix Marseille Université, C2VN, INSERM, INRAE, 13005 Marseille, France; Jean-francois.LANDRIER@univ-amu.fr; 4 Laboratoires Réunis Luxembourg S.A. 38, rue Hiehl, Z.A.C. Laangwiss, L-6131 Junglinster, Luxembourg; Bernard.Weber@labo.lu; 5 Deep Digital Phenotyping Research Unit, Department of Population Health, Luxembourg Institute of Health, 1A-B, rue Thomas Edison, L-1445 Strassen, Luxembourg; Guy.Fagherazzi@lih.lu; 6 Human Biomonitoring Research Unit, Department of Population Health, Luxembourg Institute of Health, 1A-B, rue Thomas Edison, L-1445 Strassen, Luxembourg; Brice.Appenzeller@lih.lu; 7 Nutrition and Health Research Unit, Department of Population Health, Luxembourg Institute of Health, 1A-B, rue Thomas Edison, L-1445 Strassen, Luxembourg; Torsten.Bohn@lih.lu

**Keywords:** population based study, antioxidants, adipokines, cardio metabolic health, secondary plant compounds

## Abstract

Metabolic syndrome (MetS) characteristics include chronic inflammation and elevated oxidative stress. This study assessed associations between circulating concentrations of micronutrients/phytochemicals and inflammatory/oxidative stress markers with MetS and MetS components. Adults (N = 606) from the European Health Examination Survey in Luxembourg (2013–2015) were randomly selected. We performed a multivariable logistic regression model using the least absolute shrinkage and selection operator to identify MetS-associated variables. Participants with MetS had higher concentrations of C-reactive protein (CRP), 8-iso-prostaglandin F2α, leptin, insulin, and vitamins E/A, but lower concentrations of adiponectin, beta-carotene, and oxidized low-density lipoprotein. A one-unit increase in log-CRP was associated with 51% greater odds of MetS (OR = 1.51 (95% CI: 1.16, 1.98)). Adults with a one-unit increase in log-leptin were 3.1 times more likely to have MetS (3.10 (2.10, 4.72)). Women with a one-unit increase in vitamin A were associated with 3% increased odds of MetS (1.03 (1.01, 1.05)), while those with a one-unit increase in log-adiponectin were associated with 82% decreased odds (0.18 (0.07, 0.46)). Chronic inflammation best characterized adults with MetS, as CRP, adiponectin, and leptin were selected as the main MetS determinants. Micronutrients did not seem to affect MetS, except for vitamin A in women.

## 1. Introduction

Metabolic syndrome (MetS) is defined as a constellation of different metabolic disorders (including abdominal obesity, insulin resistance, high cholesterol, and high blood pressure) that increases the risk of cardiovascular diseases (CVD), the leading cause of mortality worldwide. MetS is a global health problem, affecting nearly 25% of the world’s adult population (with regional variabilities) [1]. It is commonly associated with chronic inflammation and high levels of oxidative stress [2]. Possible causes for MetS and associated risk factors include changes in lifestyle (e.g., sedentary lifestyle or unbalanced diet), chronic exposure to environmental pollutants, and genetic predisposition. Several studies have shown an association between a healthy diet rich in fruits and vegetables and a reduction in the risk of developing chronic diseases, including CVD, type 2 diabetes (T2D), and certain types of cancer [3,4]. There is also evidence of an inverse association between fruit and vegetable consumption and MetS [5,6]. The observed health benefits can be explained by the antioxidant and anti-inflammatory properties of the combination of certain micronutrients (e.g., vitamins and minerals), phytochemicals (e.g., polyphenols, carotenoids), and some types of fermentable fiber [7], which typically relates well to the intake of fruits/vegetables from the diet [8]. In parallel, studies have observed that individuals with MetS present a reduction in blood concentrations of antioxidant vitamins and phytochemicals [9,10]. This reduction could be due to the transfer of lipophilic phytochemicals, such as carotenoids and fat soluble vitamins (A, D, E, K), from the blood into fatty tissues [11,12], causing a drop in the circulating proportion of these constituents in the bloodstream of individuals with more adipose tissue. Another possible explanation is the need for higher amounts of antioxidants in individuals with metabolic problems, which is also characterized by increased lipid peroxidation products (e.g., malondialdehyde (MDA) and oxidized low-density lipoprotein (ox-LDL)). These antioxidants may quench the increased levels of oxidative stress, such as reactive oxygen or reactive nitrogen species, causing a reduction in the concentration of circulating and/or stored antioxidants [13]. There is also the possibility that individuals with metabolic disorders have a diet characterized by low intake of such antioxidants [14]. However, there is a clear relation between low dietary intake or circulating antioxidants and increased risk of chronic disease. For instance, epidemiological studies have shown positive correlations between the intake of carotenoids and lower risk of T2D [15], and negative correlations between carotenoid plasma levels and total mortality [16] and total polyphenol intake and all-cause mortality [17]. This is supported by many in vitro and in vivo mechanistic studies showing that carotenoids can act on intracellular signaling cascades by inhibiting the formation of various downstream cytokines and/or enhancing the production of antioxidant enzymes [18]. Results observed of the possible protective effects of antioxidant vitamins are inconclusive however, with studies showing no reduction in vitamin concentrations in individuals with MetS [19], sex differences [20], and contradictory health outcomes regarding supplementation [18,21]. It should be further noted that there is a clear relation between oxidative stress and inflammation [22,23], with one potentially aggravating the other. The pro-inflammatory state in individuals with MetS is characterized by elevated concentrations of inflammatory markers, such as C-reactive protein (CRP), and alterations in the production of adipokines (proteins secreted by adipose tissue) [24]. This can be explained in part due to an excess in visceral fat characterized by dysfunctional adipocytes that secrete high concentrations of cytokines, such as leptin (pro-inflammatory role, though acting also as a hormone on satiety), and low concentrations of adiponectin (anti-inflammatory) [25]. The present study aimed to assess possible associations between circulating concentrations of micronutrients/phytochemicals, inflammatory and oxidative stress markers, and MetS in a large subsample of the population of the European Health Examination Survey in Luxembourg.

## 2. Materials and Methods

### 2.1. Study Population and Design

**Figure 1:**
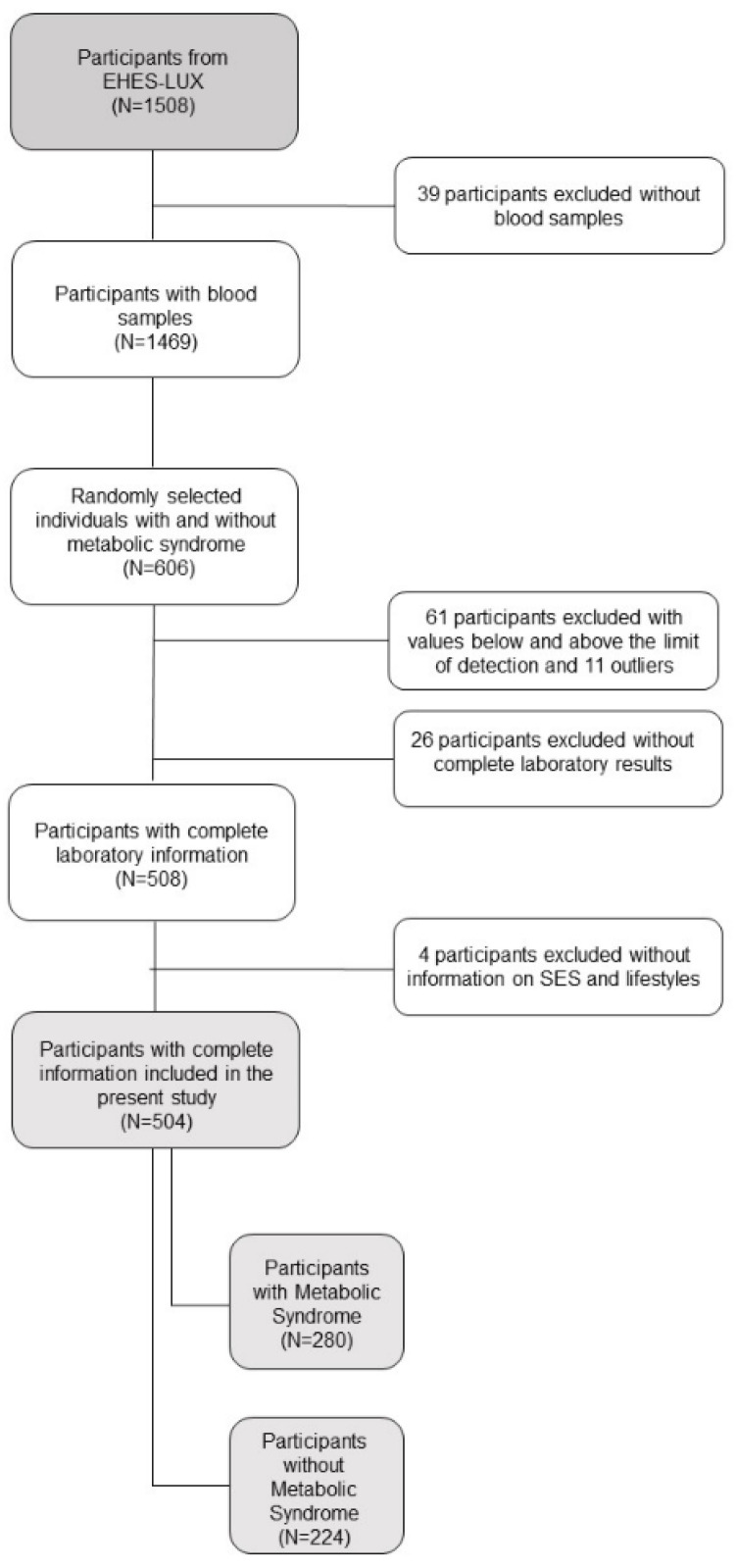
Flowchart sample selection. EHES-LUX: European Health Examination in Luxembourg; SES: socioeconomic status.

We analyzed data from 606 randomly selected individuals (315 with MetS, 291 without MetS) from the 1529 participants of the European Health Examination Survey (EHES-LUX) [26]. We selected the random sample stratified by MetS with the aim to produce a sample in which exposure varies. EHES-LUX is a cross-sectional population-based survey, representative of the general population, conducted between February 2013 and January 2015 in Luxembourg. The target sample of EHES-LUX was residents (excluding those who were living in institutions) of the Grand Duchy of Luxembourg, aged 25 to 64 years, who agreed to participate [26]. For each participant, trained nurses performed a medical examination and collected health questionnaires and biological samples (hair, blood, and urine). Examinations included blood pressure and anthropometric measurements (height, weight, waist circumference (WC), hip circumference, and thigh size), an electrocardiogram, visual acuity examination, and a spirometry. Blood samples were collected to analyze fasting blood glucose, plasma-triglycerides, plasma-cholesterol (total cholesterol, high-density lipoprotein cholesterol (HDL-C), low-density lipoprotein cholesterol (LDL-C)), and thyroid hormones. Moreover, we analyzed concentrations of selected micronutrients, markers of oxidative stress, and inflammation in the 606 selected individuals. Of these, we excluded 55 individuals with values below the limit of detection for any of the outcome measures above. We also excluded from the present analysis 16 outliers (one value of CRP > 90000 mg/mL, nine values of insulin > 2000 pmol/L, four values of F2-isoprostanes > 100.000 ng/mL, two values of folic acid > 90.8 nmol/L). Thus, 508 individuals had complete biological information. From those, 504 had complete information on socioeconomic and lifestyle characteristics (280 with MetS, i.e., cases, and 224 without MetS, i.e., controls) (Figure 1). EHES-LUX was approved by the National Ethics Committee of Luxembourg (CNER, N° 201205/07) and notified to the Commission Nationale pour la Protection des Données (CNPD). All individuals approved their participation by written informed consent.

### 2.2. Metabolic Syndrome

MetS was defined as having a WC ≥ 94 cm for men and ≥ 80 cm for women and at least the presence of two factors of the following: (1) total triglycerides ≥ 150 mg/dL or on related medication for both men and women; (2) HDL cholesterol < 40 mg/dL for men and < 50 mg/dL for women or being on related medication; (3) systolic blood pressure ≥ 130 or diastolic blood pressure ≥ 85 mm Hg or being on related medication for both men and women; and (4) fasting plasma glucose ≥ 100 mg/dL or previous diagnosis of diabetes for both men and women. We used the International Diabetes Federation definition [27].

### 2.3. Micronutrients, Markers of Oxidative Stress, and Inflammation

We analyzed circulating concentrations of 14 micronutrients/phytochemicals and inflammatory/oxidative stress markers. Cortisol, folic acid, and vitamin D (as 25 hydroxy-vitamin D) in serum aliquots (10, 25, and 20 μL, respectively) were measured by a chemiluminescent enzymatic antibody-based method (Cobas e 602, Roche) by a local commercial laboratory with an accredited method (Laboratoires Réunis, Junglinster, Luxembourg). Vitamin A (retinol) and vitamin E (alpha-tocopherol) were also measured by this laboratory from serum aliquots (Agilent 1290 Infinity II, 30 μL) based on HPLC combined with UV-Vis detection, employing the Chromosystems 34,000 kit (Chromosystems, Munich, Germany). Total carotenoids were determined following the micro-extraction procedure with heptane, similar as described by Donaldson [28]. In short, 100 µL of serum were mixed with 100 µL of methanol, including 1% butylated hydroxytoluene for protein precipitation, and then extracted with 120 and again with 70 µL of heptane. The combined fractions were measured at 450 and 470 nm spectrophotometrically (Spectra Max M2, Molecular Devices, San Jose, CA, USA) in a microcuvette (Micro Quartz Cuvette, Black, 0.7 mL, Spectrometer Cell; Science Outlet, Aliso Viejo, CA, USA). Quantification was carried out, assuming an average molecular extinction coefficient of 134.000 mol/(L * cm). Aliquots of the serum (50 µL) were measured for MDA based on the thiobarbituric acid (TBARS) assay, employing a commercial kit (10009055, Cayman Chemicals, Ann Arbout, MI, USA), based on fluorescence detection with an excitation wavelength of 525 nm and an emission wavelength of 565 nm (Spectra Max M2 spectrophotometer, Molecular Devices, San Jose, CA, USA). Total phenolics in serum aliquots were measured based on the Folin Ciocalteu assay, expressed as gallic acid equivalents. In short, 50 µL of serum and 80 µL of NaOH (2.5 M in 75% of methanol) were added to facilitate lipoprotein dissemblance. Following incubation at 37 °C for 30 min, 20 μL of 6.25 M metaphosphoric acid (MPA) was added and centrifuged (13,000× *g*, 5 min). The supernatant was removed and combined with a follow-up extraction of 250 µL of methanol (65%). Combined phases were quantified with 1:5 diluted Folin Ciocalteu reagent (Sigma Aldrich, St. Louis, MS, USA) at 750 nm in a well-plate reader (Polarstar, BMG Labtech, De Meern, The Netherlands); 8-iso-prostaglandin F2α in plasma was quantified by a direct enzyme immunoassay kit (ADI-901-091; Enzo Life Sciences AG, Villeurbanne, France). Ox-LDL in serum was measured by enzyme immunoassay (BI-20032; OLAB, Vienna, Austria). Leptin, adiponectin, CRP, and insulin in plasma (references DY398, DY1065, DY1707, DY8056, respectively) were measured using commercially available enzyme-linked immunosorbent assay kits purchased from Biotechne R&D systems (Lille, France). All quantifications were made according to the manufacturer’s specifications, after preliminary dilution tests, and based on external calibration curves.

### 2.4. Covariates

We included age, sex (men/women), and country of birth (Luxembourg, Portugal, other EU countries, other non-EU countries) as sociodemographic variables. Socioeconomic status included the variables education level (categorized into no qualification, primary education, secondary education, and tertiary education) and job status (not working, working). Lifestyle characteristics included smoking (current smoking or quitted < 12 months, non-smokers or quitted > 12 months), alcohol consumption (non-alcohol consumption, ≤6 drinks/week, >6 drinks/week), and physical activity (aerobic physical activity ≥ 150 min, aerobic physical activity < 150 min per week).

### 2.5. Statistical Data Analysis

We used means (± standard deviation) and frequencies (%) to describe the general characteristics of the sample population. Associations between MetS and potential risk factors (e.g., age, sex, smoking history, socioeconomic status) were analyzed with Pearson’s chi-squared test (χ2) or Student’s t-test as appropriate. Associations between MetS and micronutrients and markers of oxidative stress and inflammation were analyzed by the Wilcoxon test for non-parametric values. All concentrations of micronutrients/phytochemicals and markers of oxidative stress and inflammation were tested for normal distribution using the Shapiro–Wilk test of normality. Concentrations were natural log-transformed to meet residual normality. We used the least absolute shrinkage and selection operator (LASSO) [29] to select (from a total of twenty variables) the variables included in the final model by using the GLMNET package on R. The objective was to obtain the L1 penalization (λ) parameter to find the simplest model (i.e., the smallest number of coefficients) and a reduction of the bias and variance. We did a cross-validation and applied the rule of one standard error. In order to avoid over-fitting, a nonparametric bootstrapping with 1000 iterations was performed, and the selected variables were those that appeared more than 80% in all bootstrap samples. Following the selection of the variables, we calculated the odds ratios (ORs) and their 95% confidence intervals (CIs) to study the association between MetS and micronutrients, markers of oxidative stress, and inflammation. All analyses were stratified by sex. Analyses included participants with complete information for all variables. We performed a sensitivity analysis in which we replaced censored values of CRP, insulin, and leptin with the values of the detection limit divided by two. All tests were two-tailed. We considered a *p*-value of 0.05 as statistically significant. All analyses were performed using SAS 9.4 (SAS Institute Inc., Cary, NC, USA) and R (version 3.5.3).

## 3. Results

### 3.1. Participants’ Characteristics

Men had higher values of weight, BMI, and waist circumference, and presented higher levels of cardiometabolic risk factors compared to women (e.g., higher systolic and diastolic blood pressure, concentration of triglycerides, total cholesterol and fasting glucose, lower concentrations of HDL-C). Nearly half of participants included in the present study had MetS (55.6% with MetS compared to 44.4% without MetS). In our study sample (N = 504), nearly 62% of men had MetS compared to 48% of women. Men also had more MetS components (e.g., hypertriglyceridemia, hyperglycemia, and low HDL-C) compared to women (Table 1). Younger participants with higher education, with a job, and who did aerobic physical activity ≥ 150 min per week were less likely to have MetS. The same results were observed when comparing men and women, with the exception of job status for women (Supplementary Table S1).

### 3.2. Association between MetS and Markers of Inflammation, Oxidative Stress, and Micronutrients

We observed differences in blood concentrations of micronutrients/phytochemicals and inflammatory/oxidative stress markers between participants with and without MetS (Table 2). Concentrations of vitamin E and A, leptin, CRP, insulin, and 8-iso-prostaglandin F2α were higher in participants with MetS. Concentrations of adiponectin and Ox-LDL were lower in participants with MetS.

**Figure 2:**
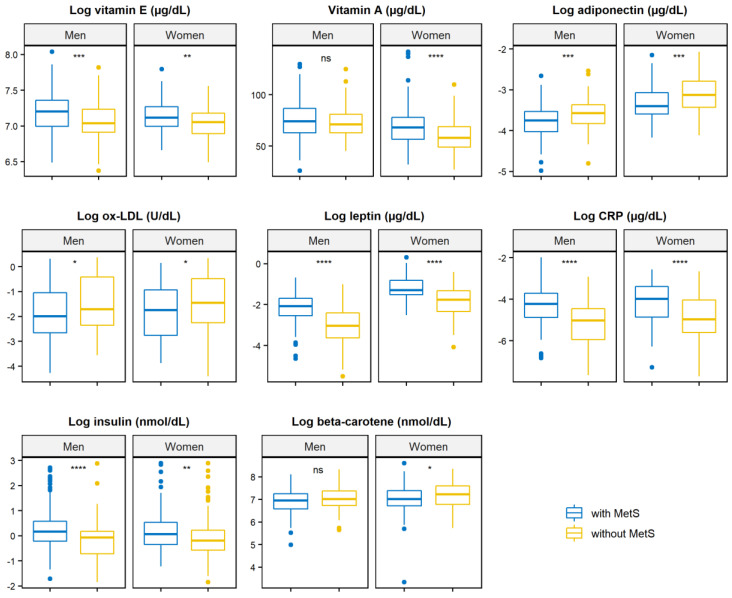
Concentrations of markers of dietary nutrient intake, inflammation, and oxidative stress by metabolic syndrome, stratified by sex. All concentrations were natural log-transformed with the exception of vitamin A. Original units prior to log-transformation were vitamin E (μg/dL), adiponectin (µg/dL), ox-LDL (U/dL), leptin (µg/dL), CRP (µg/dL), insulin (nmol/dL), and beta-carotene (nmol/dL). Ns: *p*-value > 0.05; * *p*-value ≤ 0.05; ** *p*-value ≤ 0.01; *** *p*-value ≤ 0.001; **** *p*-value ≤ 0.0001. Ox-LDL: oxidized low-density lipoprotein; CRP: c-reactive protein.

When stratified by sex (Figure 2, Supplementary Table S2), concentrations of vitamin E, leptin, CRP, and insulin were higher in men with MetS, whereas adiponectin and Ox-LDL concentrations were lower. Regarding women, concentrations of vitamins E and A, leptin, and CRP were higher for those with MetS, while concentrations of adiponectin and beta-carotene were lower.

**Figure 3:**
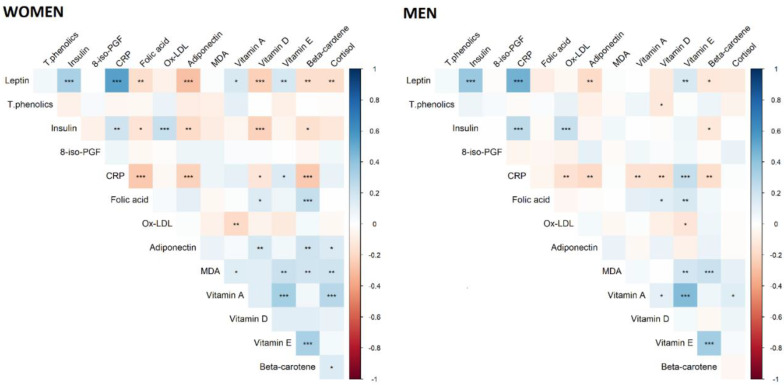
Correlations between micronutrients and markers of inflammation and oxidative stress. * *p*-value < 0.05; ** *p*-value < 0.01; *** *p*-value < 0.001. T.phenolics: total phenolics; MDA: malondialdehyde; Ox-LDL: oxidized low-density lipoprotein; CRP: c-reactive protein; 8-iso-PGF: 8-iso-prostaglandin-F2α. All concentrations were natural log-transformed with the exception of vitamin A, MDA, and cortisol.

In both men and women, we observed a positive correlation between CRP and leptin and insulin and vitamin E, and a negative correlation between CRP and adiponectin and vitamin D and beta-carotene (Figure 3). Leptin was positively correlated with insulin, CRP, and vitamin E, and negatively correlated with adiponectin and beta-carotene. We also observed a positive correlation between adiponectin and vitamin D and beta-carotene and cortisol, and a negative correlation between adiponectin and leptin and insulin and CRP in women.

Leptin, adiponectin, CRP, vitamin A, age, sex, and physical activity were selected by the LASSO logistic regression as the best predictors for MetS (Table 3). For both men and women combined, a one-unit increase in log CRP was associated with a 51% increase in the odds of having MetS (OR 1.51 (95% CI: 1.16, 1.98)), and a one-unit increase in log adiponectin was associated with a 76% decrease in the odds of having MetS (0.24 (0.13, 0.46)). Moreover, adults with a one-unit increase in log leptin were 3.10 times more likely to have MetS (3.10 (2.10, 4.72)), and a one-unit increase in vitamin A was associated with a 2% increase in the odds of having MetS (1.02 (1.01, 1.03)). We observed that, in men, a one-unit increase in log CRP was associated with a 71% increase in the odds of having MetS (1.71 (1.19, 2.51)), and men with a one-unit increase in log leptin were 3.34 times more likely to have MetS (3.34 (2.15, 5.42)). For women, a one-unit increase in log CRP was associated with a 38% increase in the odds of having MetS (1.38 (0.94, 2.06)), and a one-unit increase in vitamin A was associated with a 3% increase in the odds of having MetS (1.03 (1.01, 1.05)). Moreover, women with a one-unit increase in log leptin were 3.31 times more likely to have MetS (3.31 (1.63, 7.20)), and, in women, a one-unit increase in log adiponectin was associated with an 82% decrease in the odds of having MetS (0.18 (0.07, 0.46)).

### 3.3. MetS Components and Markers of Inflammation

When examining MetS components separately (Table 4), we observed higher odds of abdominal obesity and hyperglycemia associated with leptin in both men (15.19 (7.04, 32.79)) and (15.14 (5.64, 40.62)), respectively) and women (2.08 (1.45, 2.98) and 1.97 (1.01, 3.82), respectively). Higher odds of high blood pressure were associated with leptin (1.57 (1.08, 2.27)), and higher odds of abdominal obesity were associated with CRP (2.14 (1.33, 3.45)) only in men. In women, higher odds of abdominal obesity, hypertriglyceridemia, low HDL, and high blood pressure were associated with lower concentrations of adiponectin (0.23 (0.07, 0.70), 0.21 (0.09, 0.53), 0.27 (0.11, 0.67), and 0.31 (0.13, 0.74), respectively). We also observed that, in women, vitamin A was associated with higher odds of hypertriglyceridemia (1.04 (1.02, 1.06)), high blood pressure (1.03 (1.01, 1.05)), and hyperglycemia (1.02 (1.00, 1.04)).

## 4. Discussion

In the present study, we have provided insights into the factors that may influence the development of MetS. In particular, our results reveal the necessity to target a variety of factors, such as dietary micronutrients, inflammation, and oxidative stress. Elevated circulating concentrations of CRP, leptin, insulin, and vitamin A were all positively associated with MetS and MetS components, whereas adiponectin was inversely associated. To our knowledge, this is the first study that uses the LASSO technique to identify specifically which circulating micronutrients/phytochemicals and inflammatory/oxidative stress markers are associated with MetS.

Regarding dietary micronutrients, in our study, we included serum vitamin E, polyphenols, and carotenoids based on their frequent presence in plant-based diets [30], and vitamin A and D for their occurrence in animal-based diets [31]. Contrary to observations made in other studies [9,10], our results showed higher concentrations of vitamins E and A in individuals with MetS. In the final model, only vitamin A remained statistically significant, with a positive association observed in women only. It should be noted that, compared to other studies where participants had vitamin deficiencies, in our sample, almost all participants showed concentrations of vitamins A/E within the normal values (>20 μg/dL and >500 μg/dL, respectively). The evidence regarding the association between vitamin A and MetS, however, remains inconclusive. In a recent meta-analysis, there was no association observed between vitamin A and MetS [19]. Ford et al. [10] and Beydoun et al. [32] observed similar results to the present study, although the positive association between vitamins A/E and MetS disappeared or changed after adjusting for cholesterol and triglycerides, a result that we did not observe. In this particular case, adjusting for cholesterol and triglycerides could be justified, since vitamin E is transported through LDL-C and HDL-C. This allows us to exclude variations caused by these transporters as the underlying reason. In Western societies, individuals generally obtain vitamin A from two main dietary sources: preformed vitamin A, i.e., retinol (ca. 75% of total vitamin A intake) or provitamin A in the form of carotenoids (e.g., beta-carotene, ca. 25%) [31]. From our study, based on mainly healthy participants from the general population, vitamin A (mainly coming from animal sources, e.g., meat, dairy products, fish, and associated oils) could be an indicator of their type of diet. This would explain why participants with MetS showed higher concentrations of vitamin A, since their diet might be rich in animal sources and low in fruit- and vegetable-derived carotenoids. Further on this hypothesis and the results observed in other studies, we also observed lower serum levels of beta-carotene in individuals with MetS [19]. Studies have shown that a diet rich in carotenoids can have a protective function in the development of chronic diseases [10,15,19]. This effect could be mediated by carotenoids acting as antioxidants, modulating transcription factors while also regulating inflammation [18]. However, in our study, beta-carotene was not selected in our final model. As for carotenoids, studies have observed that higher intakes of polyphenols are associated with reduced cardiometabolic risk factors [33]. In our study, we did not observe this association, although it is possible that i) our analytical test was not specific enough for dietary polyphenols, since it also detected other reduced compounds, and ii) the low bioavailability of many polyphenols impeded clear findings [33].

Our results emphasize the inflammatory response related to MetS, since biomarkers of inflammation, such as CRP, adiponectin, and leptin, were selected in our final model. Levels of CRP are increased in response to inflammation and are considered an indicator of coronary risk and metabolic problems [34]. On the other hand, levels of adiponectin (secreted by adipose tissue) are reduced in response to chronic inflammation [35]. The role of adiponectin was mainly observed in women. Sex-based differences in the strength of the association between adiponectin and MetS could be explained by the fact that women had higher concentrations of circulating adiponectin compared to men. These sex differences seemed to be related to body composition and fat distribution, since women usually have less visceral adipose tissue and more subcutaneous adipose tissue than men [36]. Both CRP and adiponectin are considered as key players in the development of MetS, characterized by an imbalance of pro- and anti-inflammatory response in favor of the first. Furthermore, we observed that leptin had the strongest association with MetS, although the main association was observed with abdominal obesity as expected, since concentrations of leptin are correlated with total body fat [37]. This association was observed in both men and women regardless of concentrations being higher in women than men. Leptin was also associated with high blood pressure in men and hyperglycemia in both men and women. Cross-sectional and prospective studies have observed an association between leptin concentrations and MetS independent of obesity [38,39]. Leptin has different biological roles, including energy balance and endocrine function [40]. Studies have observed that a dysfunctional adipose tissue produces adipokines (e.g., adiponectin and leptin) in abnormal concentrations and is associated with cardiometabolic problems [37]. Leptin resistance has also been identified as a risk factor associated with MetS, similar to insulin resistance [41]. Insulin concentrations were also higher in adults with MetS, though in the final model the association was not statistically significant. Although insulin resistance is a characteristic of individuals with MetS, MetS may appear without the presence of elevated insulin concentrations [42]. This could explain why insulin was selected in our model, but its association was not statistically significant.

While inflammation was related to MetS, no marker of oxidative stress (including OxLDL, MDA, or 8-iso-prostaglandin F2α) was associated with MetS or its components. This contrasts with previous findings that have associated these markers with MetS [43,44]. It is possible that the limited differences in dietary status and the overall relatively healthy population prevented an imbalance in oxidative stress homeostasis.

Limitations of the present study include the cross-sectional design of our study, which does not allow drawing conclusions about causality. Moreover, we only analyzed data from participants with complete measurements, and therefore lost information by excluding participants with incomplete data. Strengths include the individual and objective measurement of a large number of risk factors. In addition, the LASSO selection technique allowed us to select the variables that best explained the association.

## 5. Conclusions

The present study contributes to a better understanding of the key determinants (selected from a large set of possible factors of MetS). Oxidative stress, inflammation, and nutritional status are among several factors that may influence the onset of MetS, and this study emphasizes that chronic inflammation appears to best characterize individuals with MetS.

## Figures and Tables

**Table 1:** Cardiometabolic characteristics of the sample stratified by sex (N = 504).

	Total (N = 504)	Men (N = 272)	Women (N = 232)	*p*-Value ^1^
	Mean ± SD, N (%)	Mean ± SD, N (%)	Mean ± SD, N (%)	
**Anthropometric characteristics**				
Weight, kg	81.61 ± 18.47	88.37 ± 17.10	73.67 ± 16.83	<0.0001
BMI, kg/m^2^	28.34 ± 5.670	28.71 ± 4.830	27.90 ± 6.510	0.12
Waist, cm	96.24 ± 15.19	101.0 ± 13.32	90.62 ± 15.36	<0.0001
Hip size, cm	104.3 ± 11.06	102.8 ± 9.130	106.0 ± 12.78	<0.01
**Cardiometabolic risk factors**				
Systolic blood pressure, mm Hg	127.1 ± 16.63	132.3 ± 14.41	121.0 ± 17.02	<0.0001
Diastolic blood pressure, mm Hg	81.89 ± 11.14	84.10 ± 10.93	79.28 ± 10.83	<0.0001
Triglycerides, mg/dL	134.8 ± 81.65	160.9 ± 91.56	104.1 ± 54.18	<0.0001
HDL-C, mg/dL	48.22 ± 13.53	42.81 ± 10.58	54.56 ± 13.86	<0.0001
Total cholesterol, mg/dL	203.6 ± 38.53	204.6 ± 40.43	202.3 ± 36.24	0.50
Glucose, mg/dL	101.5 ± 21.28	104.8 ± 25.73	97.53 ± 13.45	<0.0001
HbA1c, mol/mol	38.51 ± 6.840	38.72 ± 7.960	38.27 ± 5.250	0.45
Metabolic syndrome	280 (55.56)	169 (62.13)	111 (47.84)	<0.01
**Metabolic syndrome components**				
Abdominal obesity ^2^	350 (69.44)	185 (52.86)	165 (47.14)	0.45
Hypertriglyceridemia ^3^	230 (45.63)	153 (66.52)	77 (33.48)	<0.0001
Low HDL-C ^4^	219 (43.35)	148 (67.58)	71 (32.42)	<0.0001
High blood pressure ^5^	265 (52.68)	145 (54.72)	120 (45.28)	0.76
Hyperglycemia ^6^	289 (57.46)	182 (62.98)	107 (37.02)	<0.0001

N: number; SD: standard deviation; BMI: body mass index; HDL-C: high-density lipoprotein cholesterol; HbA1c: glycated hemoglobin A1c. ^1^ Pearson’s chi-squared test for categorical variables, t-test for continuous variables. ^2^ Waist circumference ≥ 94 cm for men and ≥ 80 cm for women. ^3^ Total triglycerides ≥ 150 mg/dL or on related medication. ^4^ HDL cholesterol < 40 mg/dL for men and < 50 mg/dL for women or on related medication. ^5^ Systolic blood pressure ≥ 130 or diastolic blood pressure ≥ 85 mm Hg or on related medication. ^6^ Fasting plasma glucose ≥ 100 mg/dL or self-reported based on a physician diagnosis.

**Table 2:** Concentration of micronutrients and circulating markers of inflammation and oxidative stress by metabolic syndrome (N = 504).

	Metabolic Syndrome (N = 280)					Non Metabolic Syndrome (N = 224)					
	Median	Q25	Q75	Min	Max	Median	Q25	Q75	Min	Max	*p*-Value ^1^
**Markers of dietary intake**											
Vitamin D, µg/dL	0.213	0.147	0.273	0.046	0.710	0.224	0.156	0.282	0.062	0.536	0.32
Vitamin E, μg/dL	1274	1087	1538	657.0	3100	1154	993.0	1327	587.0	2487	<0.0001
Vitamin A, μg/dL	71.50	59.50	82.00	26.00	142.0	65.00	53.00	75.00	27.00	125.0	<0.0001
Folic acid, nmol/dL	122.0	89.50	178.0	29.00	546.0	132.0	93.50	180.0	26.00	686.0	0.36
Beta-carotene, nmol/dL	1078	751.0	1510	28.00	5459	1220	851.0	1822	284.0	4268	<0.01
Total phenolics, mmol/dL	1072	1006	1144	785.3	1384	1065	1014	1125	781.2	1351	0.66
**Markers of oxidative stress**											
MDA, μM	1.101	0.847	1.389	0.171	2.605	1.102	0.807	1.343	0.132	2.520	0.49
Ox-LDL, U/dL	0.157	0.067	0.374	0.014	1.368	0.231	0.100	0.647	0.012	1.453	<0.001
8-Iso-prostaglandin-F2α, µg/dL	0.019	0.009	0.086	0.002	0.412	0.017	0.009	0.067	0.001	0.317	0.34
**Marker of stress**											
Cortisol, nmol/dL	4153	3243	5150	112.0	12980	4152	3346	5202	1025	16240	0.95
**Markers of inflammation**											
CRP, µg/dL	0.016	0.008	0.028	0.001	0.138	0.007	0.003	0.014	0.000	0.070	<0.0001
Adiponectin, µg/dL	0.027	0.020	0.036	0.007	0.117	0.034	0.025	0.047	0.008	0.132	<0.001
**Markers of energy metabolism and hormones**											
Leptin, µg/dL	0.176	0.112	0.268	0.010	1.365	0.096	0.045	0.192	0.004	0.667	<0.0001
Insulin, nmol/dL	1.148	0.760	1.761	0.180	17.97	0.919	0.540	1.228	0.157	18.00	<0.0001

N: number; Q: quartile; MDA: malondialdehyde; Ox-LDL: oxidized low-density lipoprotein; CRP: c-reactive protein; ^1^ Wilcoxon test.

**Table 3:** Variables selected by the LASSO logistic regression, measuring the association between metabolic syndrome and micronutrients and markers of inflammation and oxidative stress (N = 504).

	Metabolic Syndrome		
	Men	Women	Total
	OR (95% CI)	OR (95% CI)	OR (95% CI)
Vitamin A, μg/dL	-	1.03 (1.01, 1.05)	1.02 (1.01, 1.03)
CRP, μg/dL	1.71 (1.19, 2.51)	1.38 (0.94, 2.06)	1.51 (1.16, 1.98)
Adiponectin, μg/dL	-	0.18 (0.07, 0.46)	0.24 (0.13, 0.46)
Leptin, μg/dL	3.34 (2.15, 5.42)	3.31 (1.63, 7.22)	3.10 (2.10, 4.72)
Insulin, nmol/dL	-	-	1.24 (0.90, 1.73)
Age, years	1.08 (1.04, 1.12)	1.13 (1.09, 1.18)	1.10 (1.08, 1.14)
Aerobic Physical activity (≥150 min/week vs. <150 min/week)	0.41 (0.21, 0.77)	0.28 (0.11, 0.63)	0.32 (0.19, 0.54)
Sex (women vs. men)	-	-	0.26 (0.13, 0.51)

OR: odds ratio; 95% CI: 95% confidence interval; LASSO: least absolute shrinkage and selection operator; CRP: c-reactive protein. All concentrations were natural log-transformed.

**Table 4:** Variables selected by the LASSO logistic regression, measuring the association between metabolic syndrome components and micronutrients and markers of inflammation and oxidative stress (N = 504).

	Men, N = 272	Women, N = 231	Total, N = 503
	OR (95% CI)	OR (95% CI)	OR (95% CI)
**Abdominal obesity** ^1^			
Vitamin A, μg/dL	-	1.01 (0.98, 1.03)	1.00 (0.98, 1.01)
CRP, μg/dL	2.14 (1.33, 3.45)	1.13 (0.74, 1.73)	1.43 (1.06, 1.94)
Adiponectin, μg/dL	-	0.23 (0.07, 0.70)	0.29 (0.13, 0.64)
Leptin, μg/dL	15.2 (7.04, 32.8)	15.1 (5.64, 40.6)	13.5 (7.44, 24.6)
Insulin, nmol/dL	-	-	1.15 (0.77, 1.73)
Age, years	1.07 (1.03, 1.12)	1.12 (1.07, 1.18)	1.10 (1.06, 1.14)
Aerobic Physical activity (≥150 min/week vs. <150 min/week)	0.66 (0.29, 1.52)	0.38 (0.15, 0.99)	0.48 (0.26, 0.89)
Sex (women vs. men)	-	-	0.18 (0.07, 0.47)
**Hypertriglyceridemia ^2^**			
Vitamin A, μg/dL	-	1.04 (1.02, 1.06)	1.04 (1.02, 1.05)
CRP, μg/dL	1.27 (0.95, 1.70)	1.26 (0.84, 1.87)	1.33 (1.04, 1.70)
Adiponectin, μg/dL	-	0.21 (0.09, 0.53)	0.26 (0.15, 0.47)
Leptin, μg/dL	1.33 (0.95, 1.86)	1.64 (0.83, 3.27)	1.27 (0.92, 1.76)
Insulin, nmol/dL	-	-	1.14 (0.87, 1.51)
Age, years	1.05 (1.02, 1.07)	1.10 (1.06, 1.15)	1.06 (1.04, 1.09)
Aerobic Physical activity (≥150 min/week vs. <150 min/week)	0.75 (0.44, 1.29)	0.61 (0.26, 1.45)	0.64 (0.40, 1.04)
Sex (women vs. men)	-	-	0.51 (0.28, 0.93)
**Low HDL-C ^1^**			
Vitamin A, μg/dL	-	1.00 (0.98, 1.02)	1.00 (0.99, 1.01)
CRP, μg/dL	1.34 (0.98, 1.84)	0.87 (0.62, 1.23)	1.08 (0.86, 1.37)
Adiponectin, μg/dL	-	0.27 (0.11, 0.67)	0.33 (0.19, 0.59)
Leptin, μg/dL	1.08 (0.75, 1.55)	1.70 (0.95, 3.04)	1.17 (0.85, 1.61)
Insulin, nmol/dL	-	-	1.07 (0.82, 1.41)
Age, years	1.00 (0.97, 1.03)	1.08 (1.04. 1.12)	1.03 (1.01, 1.06)
Aerobic Physical activity (≥150 min/week vs. <150 min/week)	0.78 (0.43, 1.39)	0.50 (0.24, 1.05)	0.66 (0.42, 1.04)
Sex (women vs men)	-	-	2.13 (1.14, 3.98)
**High blood pressure ^1^**			
Vitamin A, μg/dL	-	1.03 (1.01, 1.05)	1.02 (1.01, 1.04)
CRP, μg/dL	1.37 (0.98, 1.90)	1.47 (1.01, 2.14)	1.30 (1.02, 1.65)
Adiponectin, μg/dL	-	0.31 (0.13, 0.74)	0.45 (0.25, 0.81)
Leptin, μg/dL	1.57 (1.08, 2.27)	1.07 (0.59, 1.94)	1.28 (0.93, 1.78)
Insulin, nmol/dL	-	-	1.52 (1.13, 2.05)
Age, years	1.07 (1.03, 1.10)	1.11 (1.07, 1.16)	1.09 (1.07, 1.12)
Aerobic Physical activity (≥150 min/week vs. <150 min/week)	0.47 (0.26, 0.85)	0.57 (0.26, 1.22)	0.50 (0.31, 0.80)
Sex (women vs. men)	-	-	0.41 (0.21, 0.79)
**Hyperglycemia ^1^**			
Vitamin A, μg/dL	-	1.02 (1.00, 1.04)	1.01 (1.00, 1.03)
CRP, μg/dL	0.93 (0.69, 1.25)	1.08 (0.74, 1.60)	1.02 (0.81, 1.28)
Adiponectin, μg/dL	-	0.44 (0.18, 1.05)	0.73 (0.43, 1.25)
Leptin, μg/dL	2.08 (1.45, 2.98)	1.97 (1.01, 3.82)	1.97 (1.42, 2.73)
Insulin, nmol/dL	-	-	1.14 (0.87, 1.50)
Age, years	1.04 (1.01, 1.07)	1.10 (1.06, 1.14)	1.06 (1.04, 1.08)
Aerobic Physical activity (≥150 min/week vs. <150 min/week)	0.84 (0.48, 1.46)	0.51 (0.23, 1.15)	0.72 (0.46, 1.13)
Sex (women vs. men)	-	-	0.23 (0.13, 0.43)

OR: odds ratio; 95% CI: 95% confidence interval; LASSO: least absolute shrinkage and selection operator; CRP: c-reactive protein. All concentrations were natural log-transformed, except for vitamin A, triglycerides, and cholesterol. ^1^ Models adjusted for total cholesterol and triglycerides; ^2^ Model adjusted for total cholesterol.

## Supplementary Materials

The following are available online at https://www.mdpi.com/2072-6643/13/1/5/s1, Table S1: Participants’ sociodemographic, economic, and lifestyle characteristics by metabolic syndrome, stratified by sex (N = 504); Table S2: Concentration of micronutrients and markers of inflammation and oxidative stress by metabolic syndrome, stratified by sex (N = 504).
