# Supplementary material for: Micronutrients and Markers of Oxidative Stress and Inflammation Related to Cardiometabolic Health: Results from the EHES-LUX Study
Source: Nutrients. 2020 Dec 22;13(1):5. doi: 10.3390/nu13010005 (PMC7822009; doi:10.3390/nu13010005)
Supplement: Supplementary file 1 [file nutrients-13-00005-s001.pdf]

Supplementary material

Table S1. Participants' sociodemographic, economic and lifestyle characteristics by metabolic syndrome, stratified by sex (N=504).

	Total (N = 504)			Men (N= 272)			Women (N=232)		
	MetS N (%)	Non MetS N (%)	P-value[1]	MetS N (%)	Non MetS N (%)	P-value[1]	MetS N (%)	Non MetS N (%)	P-value[1]
Age, years			<0.0001			<0.0001			<0.0001
25-34	15 (5.36)	57 (25.45)		10 (5.92)	30 (29.13)		5 (4.50)	27 (22.31)	
35-44	67 (23.93)	82 (36.61)		47 (27.81)	35 (33.98)		20 (18.02)	47 (38.84)	
45-54	93 (33.21)	45 (20.09)		54 (31.95)	19 (18.45)		39 (35.14)	26 (21.49)	
55-64	105 (37.50)	40 (17.86)		58 (34.32)	19 (18.45)		47 (42.34)	21 (17.36)	
Country of birth			0.28			0.14			0.19
Luxembourg	131 (46.79)	97 (43.30)		90 (53.25)	41 (39.81)		41 (36.94)	56 (46.28)	
Portugal	58 (20.71)	37 (16.52)		31 (18.34)	20 (19.42)		27 (24.32)	17 (14.05)	
Other EU countries	67 (23.93)	63 (28.13)		36 (21.30)	31 (30.10)		31 (27.93)	32 (26.45)	
Other non-EU countries	24 (8.57)	27 (12.05)		12 (7.10)	11 (10.68)		12 (10.81)	16 (13.22)	
Education level			<0.0001			0.01			<0.0001
No qualification/Primary	99 (35.36)	44 (19.64)		49 (28.99)	23 (22.33)		50 (45.05)	21 (17.36)	
Secondary	103 (36.79)	77 (34.38)		65 (38.46)	28 (27.18)		38 (34.23)	49 (40.50)	
Tertiary	78 (27.86)	103 (45.98)		55 (32.54)	52 (50.49)		23 (20.72)	51 (42.15)	
Job status			<0.01			0.11			<0.01
Not working	91 (32.50)	48 (21.43)		40 (23.67)	16 (15.53)		51 (45.95)	32 (26.45)	
Working	189 (67.50)	176 (78.57)		129 (76.33)	87 (84.47)		60 (54.05)	89 (73.55)	
Smoking			0.74			0.57			0.41
Non-smokers or quit >12 months	206 (73.57)	167 (74.55)		123 (72.78)	71 (68.93)		83 (74.77)	96 (79.34)	
Current smoking or quit <12 months	74 (26.43)	56 (25.00)		46 (27.22)	31 (30.10)		28 (25.23)	25 (20.66)	
Alcohol consumption			0.12			0.68			0.37

Non-alcohol consumption	103 (36.79)	90 (40.18)		36 (21.30)	22 (21.36)		67 (60.36)	68 (56.2)	
≤ 6 drinks/week	75 (26.79)	72 (32.14)		51 (30.18)	36 (34.95)		24 (21.62)	36 (29.75)	
> 6 drinks/week	101 (36.07)	62 (27.68)		82 (48.52)	45 (43.69)		19 (17.12)	17 (14.05)	
Aerobic Physical activity			<0.0001			<0.0001			<0.01
< 150 min/week	222 (79.29)	132 (58.93)		128 (75.74)	52 (50.49)		94 (84.68)	80 (66.12)	
≥ 150 min/week	58 (20.71)	92 (41.07)		41 (24.26)	51 (49.51)		17 (15.32)	41 (33.88)	

N: number; MetS : metabolic syndrome. [1]Pearson Chi2 test.

Table S2. Concentration of micronutrients and markers of inflammation and oxidative stress by metabolic syndrome, stratified by sex (N=504).

	Metabolic Syndrome (N=280)					Non Metabolic Syndrome (N=224)					
	Median	Q25	Q75	Min	Max	Median	Q25	Q75	Min	Max	P-Value[1]
Men (N=169/ 103)											
Markers dietary intake											
Vitamin D, μg/dL	0.203	0.137	0.263	0.046	0.505	0.192	0.128	0.258	0.070	0.432	0.79
Vitamin E, μg/dL	1344	1092	1571	657	3100	1140	1004	1385	587.0	2487	<0.001
Vitamin A, μg/dL	74.00	63.00	86.70	26.00	130.0	71.00	63.00	81.00	45.00	125.0	0.28
Folic acid, nmol/dL	113.0	88.00	163.0	29.00	546.0	121.0	76.00	162.0	26.00	314.0	0.73
Beta-carotene, nmol/dL	1049	723.0	1418	147.0	3318	1120	837.0	1616	284.0	4154	0.06
Total phenolics, mmol/dL	1074	1005	1137	785.3	1370	1076	1010	1130	842.6	1315	0.82
Markers of oxidative stress											
MDA, μM	1.102	0.850	1.420	0.171	2.605	1.107	0.810	1.358	0.132	2.524	0.55
Ox-LDL, U/dL	0.136	0.070	0.353	0.014	1.368	0.180	0.095	0.677	0.035	1.453	0.01
8-Iso-prostaglandin-F2α, μg/dL	0.017	0.008	0.092	0.002	0.404	0.016	0.010	0.066	0.001	0.288	0.84
Marker of stress											
Cortisol, nmol/dL	4140	3325	4890	112.0	7794	4016	3426	4823	1025	7169	0.77
Markers inflammation											
CRP, μg/dL	0.015	0.008	0.024	0.001	0.138	0.007	0.003	0.012	0.000	0.054	<0.0001

Adiponectin, μg/dL	0.023	0.018	0.029	0.007	0.070	0.028	0.022	0.035	0.008	0.079	<0.001
Markers of energy metabolism and hormones											
Leptin, μg/dL	0.126	0.078	0.183	0.010	0.509	0.048	0.026	0.092	0.004	0.366	<0.0001
Insulin, nmol/dL	1.187	0.808	1.788	0.180	15.030	0.937	0.481	1.200	0.158	17.79	<0.0001
Women (N=111/121)											
Markers dietary intake											
Vitamin D, μg/dL	0.224	0.164	0.279	0.091	0.710	0.234	0.177	0.308	0.062	0.536	0.33
Vitamin E, μg/dL	1230	1081	1441	780.0	2423	1158	986.0	1310	661.0	1918	<0.01
Vitamin A, μg/dL	68.00	56.00	78.00	32.00	142.0	58.00	49.00	69.00	27.00	110.0	<0.0001
Folic acid, nmol/dL	132.0	94.00	204.0	35.00	440.0	141.0	102.0	202.0	40.00	686.0	0.38
Beta-carotene, nmol/dL	1120	808.0	1616	28.00	5459	1375	879.0	2013	312.0	4268	0.03
Total phenolics, mmol/dL	1062	1008	1149	882.8	1384	1061	1016	1121	781.2	1351	0.82
Markers of oxidative stress											
MDA, μM	1.101	0.838	1.382	0.431	2.331	1.090	0.791	1.339	0.348	2.487	0.73
Ox-LDL, U/dL	0.175	0.061	0.405	0.021	1.160	0.235	0.106	0.619	0.012	1.407	0.01
8-Iso-prostaglandin-F2α, μg/dL	0.023	0.009	0.084	0.002	0.412	0.017	0.009	0.068	0.003	0.317	0.28
Marker of stress											
Cortisol, nmol/dL	4275	3142	5567	1190	12980	4327	3164	5377	1263	16240	0.99
Markers inflammation											
CRP, μg/dL	0.019	0.008	0.034	0.001	0.077	0.007	0.004	0.018	0.000	0.070	<0.0001
Adiponectin, μg/dL	0.033	0.027	0.046	0.015	0.117	0.044	0.034	0.062	0.016	0.127	<0.0001
Markers of energy metabolism and hormones											
Leptin, μg/dL	0.275	0.218	0.442	0.080	1.365	0.171	0.096	0.267	0.018	0.667	<0.0001
Insulin, nmol/dL	1.070	0.700	1.720	0.290	17.970	0.823	0.563	1.243	0.157	18.000	<0.01

N: number; Q: quartile; MDA: malondialdehyde; Ox-LDL: oxidized low-density lipoprotein; CRP: c-reactive protein; [1]Wilcoxon test
